# LFastqC: A lossless non-reference-based FASTQ compressor

**DOI:** 10.1371/journal.pone.0224806

**Published:** 2019-11-14

**Authors:** Sultan Al Yami, Chun-Hsi Huang

**Affiliations:** 1 Computer Science and Engineering, University of Connecticut, Storrs, Connecticut, United States of America; 2 Computer Science and Information System, Najran University, Najran, Saudi Arabia; University of Helsinki, FINLAND

## Abstract

The cost-effectiveness of next-generation sequencing (NGS) has led to the advancement of genomic research, thereby regularly generating a large amount of raw data that often requires efficient infrastructures such as data centers to manage the storage and transmission of such data. The generated NGS data are highly redundant and need to be efficiently compressed to reduce the cost of storage space and transmission bandwidth. We present a *lossless*, *non-reference-based* FASTQ compression algorithm, known as LFastqC, an improvement over the LFQC tool, to address these issues. LFastqC is compared with several state-of-the-art compressors, and the results indicate that LFastqC achieves better compression ratios for important datasets such as the LS454, PacBio, and MinION. Moreover, LFastqC has a better compression and decompression speed than LFQC, which was previously the top-performing compression algorithm for the LS454 dataset. LFastqC is freely available at https://github.uconn.edu/sya12005/LFastqC.

## Introduction

Next-generation sequencing (NGS) technologies have accelerated genomic research, thereby producing significant amount of data at a fast pace and low cost. However, the storage technology is evolving at a much slower pace compared with the NGS technologies, thereby posing challenges for data storage. Data centers are often used as a solution, while incurring considerable costs for storage space and transmission bandwidth. The time required for data transmission over network can be reduced by compressing the highly redundant genomics data. Most data centers use generic compressors, such as the *gzip* and *bzip2*. However, these are not ideal solutions for compressing NGS data since both were designed for general-purposes compression and have been shown to perform inadequately when compressing genomic data. Therefore, it is important for researchers to have an appropriate tool that is specifically developed for NGS data compression. The NGS-generated data are stored in a FASTQ format; FASTQ files comprise millions-to-billions of records, each of which containing the following four lines:

- Line 1 stores a sequence identifier and begins with @.
- Line 2 represents the read sequence.
- Line 3 begins with the + character, optionally followed by the same sequence identifier as Line 1.
- Line 4 represents the quality score value for the sequence in Line 2 and has the same length as Line 2.

As indicated above, the NGS produces DNA sequences and a corresponding quality score value for each base. This value is the probability that the corresponding base call is incorrect. These error values are then converted into integers. The *Ewing and Green algorithm* [1] transformed these error values into *PHRED quality scores* (QS) [2], which can be used to characterize the quality of DNA sequences. The algorithm uses the following equation to calculate QS, *i*.*e*. *Q* = −10 log_10_*p*. Then, the resulting values are shortened to fit within 0–93; this range represents the values for the base score in the ASCII form. Each integer is then incremented by 33 so that all values are in the range of printable ASCII characters.

In recent years, DNA sequence compression has been actively investigated, and many tools have been specifically developed for this application. One example is the *MFCompress* [3], which uses the *finite-context model* for compression. The finite-context model is a probabilistic model that utilizes a probability distribution and estimates the probability of the next symbol in a sequence based on the k previous symbols. MFCompress encodes the DNA sequences using multiple competing finite-context models [4], as well as arithmetic encoding. Another algorithm is the *DNA-COMPACT*, which is based on a *pattern-aware* contextual modeling technique [5]. This algorithm exploits complementary contextual models and works in two phases. In the first phase, the algorithm searches for the exact repeats and palindromes and then represents them by a compact quadruplet. In the second phase, the algorithm introduces the non-sequential contextual models to utilize the DNA sequence characteristics, and then a *logistic regression model* is used to synthesize the predictions of these models. Another algorithm is the *SeqCompress* [6], which is based on a statistical model and arithmetic coding and can efficiently compress both repetitive and non-repetitive sequences.

## Related work

A similar problem to DNA sequence compression is the compression of FASTQ data, which consists of a DNA sequence of varying-length, a quality score, and an identifier. Compressing FASTQ requires new techniques that compress each stream of FASTQ independently and efficiently. Recently, several domain-specific data compressors have been developed for NGS data compression. *DSRC* [7,8], for example, is a fast FASTQ data compressor designed for industry. *Quip* [9] is another tool that uses a de novo assembly algorithm and was the first assembly-based compressor. Another tool is the *fqzcomp* [10], which was the winner of the *Sequence Squeeze competition* organized by the Pistoia Alliance. *Fastqz* is another compressor which uses context modeling to reduce the data volume to maximize the compression gain [10]. *LFQC* [11], which was developed recently and yielded the best compression ratio on the LS454 and SOLEXA datasets. Another recently developed tool is the *FaStore*, which is optimized to handle files generated by sequencing machines that generate a large number of short reads at a low sequencing error rate [12]. *SPRING* is another FASTQ compressor which was developed recently and provides high compression and decompression speed [13]. However, some of the previous algorithms have failed to compress some datasets due to the lack of support for reading variable-length read or space encoding, such as the *color space encoding* used in SOLiD.

This study presents the *LFastqC*, a lossless non-reference-based compression algorithm, which is an extension of the LFQC but performs better when using only the quality score as a length reference for the sequence stream. The algorithm is discussed and evaluated in the following sections. The compression result is compared with results from other methods that also adopt a lossless non-reference-based compression technique.

## Materials and methods

### FASTQ data compression

NGS files are stored in the FASTQ format, which typically consist of millions-to-billions of records with four lines each. Line 1 represents the record identifier, Line 2 stores the record nucleotide, Line 3 represents a constant “+” optionally followed by record identifier, and Line 4 represents the corresponding quality scores for the nucleotide sequences from Line 2. Each line comprises four different alphabet sets and has its own structure; therefore, several existing FASTQ compressors compress each line separately. The proposed algorithm follows the literature by splitting the FASTQ file into three data streams; each stream is then preprocessed independently for compression. A regular data compressor and a special-purpose Fasta file compressor, *i*.*e*. lpaq8 (http://mattmahoney.net/dc/#lpaq) and MFCompress (http://bioinformatics.ua.pt/software/mfcompress), are used at the compression stages.

### lpaq8 compressor

lpaq8 is a part of the PAQ series, which are lossless data-compression archivers that adopt a context mixing algorithm. These algorithms divided the compressor into a predictor and an arithmetic coder, and work just like *prediction by partial matching* (PPM). However, they are different from PPM in two ways. First of all, context mixing algorithms predict the next symbol by computing a weighted combination of probability estimates from many models on different contexts. Secondly, context mixing algorithms use many models, unlike PPM which uses a single model. Context mixing algorithms yield the best performance on many benchmarks in terms of compression ratio. These benchmarks vary in size and alphabet, rendering lpaq8 the best option when compressing quality score and read identifiers.

### MFCompress compressor

MFCompress relies on finite-context models, which is probabilistic and works by estimating the probability of the next symbol of the source based on the frequencies of the symbols that occurred before the current symbol. MFCompress uses multiple competing finite-context models to compress the DNA sequences and uses a single finite-context models to compress the file header. MFCompress compresses Fasta files efficiently in term of time and compression gain, making it suitable for compressing the DNA sequence stream in a FASTQ file.

The following subsections explain how LFastqC pre-processes each stream before sending it to the corresponding compression tool.

### Identifier compression

The main goal of the identifier field is to uniquely identify the read. The reads are identified using an integer value, but the identifiers have more information than what is needed to identify each read. For example, the identifier field contains the instrument’s name, run identifier, flow cell identifier, and tile coordinates. Most of this information is the same for every read. This redundancy increases the file size, but it can also be utilized to achieve better compression of the identifier stream.

The identifier fields can be classified as one of three types: fields with data that do not change over the whole records, fields with the same data value over a set of consecutive records, and fields with integer values that are either incremented or decremented over consecutive records. LFastqC takes this information into consideration when preprocessing this stream. The algorithm first scans the identifier for one of these delimiters: a dot (.), underscore (_), space (), hyphen (–), colon (:), equal sign (=), or slash (/). It then splits the identifiers into fields based on these delimiters. This process leads to the creation of new files with one column (the field column) and N rows, where N is the number of records.

The following example explains how the algorithm splits a record identifier using the following record identifier:

@SRR001471.1E96DJWM01D47CSlength=79

The algorithm first returns the identifier’s delimiters, which are the following in this case: a dot (.), a space (), a space (), and then an equal sign (=). Thus, the identifier is split into five fields:

- @SRR001471
- 1
- E96DJWM01D47CS
- length
- 79

At this point, some FASTQ compressors in the literature add a layer of compression by compressing each field using one of the following compression techniques: *delta encoding*, *run length encoding*, or *reversing the fields* (reading them from right to left) for further compression. We observed that this compression layer did not improve the compression ratio in general, but increased the running time altogether. Instead, for our algorithm feeds the identifier fields to the lpaq8 compressor at this point, which is a standard context mixing compression algorithm. We use lpaq8 with option “9” which yielded the best compression ratio.

### Sequence compression

The nucleotide sequences are arranged in a small string of five alphabetic characters, namely A, C, G, T, and N. The N base contains unknown nucleotides and always has “!” as its corresponding quality score, which indicates the lowest probability and is equal to zero. Some FASTQ algorithms eliminate “N” in the record sequences or “!” in the record quality score because they can be easily reconstructed from one another. Our algorithm does not follow this approach as we simply use the quality score as a read-length reference.

Some other datasets use color space encoding, which means that the read sequence has more than five characters. The color-space read sequence starts with any of A, C, G, or T, followed by numbers 0–3, which represent the relationship between the current base and the previous one. Our algorithm supports these datasets because it uses MFCompress, a FASTA and multi-FASTA special-purpose compressor that accepts FASTA files with more than five characters. To compress the record sequences, our algorithm first converts the stream into a single FASTA file by adding the header of the first sequence as the first line, then deleting all sequence reads’ new lines to get a long single sequence read. LFastqC then feeds the converted stream to MFCompress for compression. We use MFCompress with a parameter of -3 and obtain the best compression ratio.

### Quality score compression

Each record has a quality score, which has the same length as the record sequence. We use it as a length reference for the sequence reconstruction since we converted the record sequence to a single FASTA file. According to the literature, there is a correlation between any quality score and the score at the preceding position. This correlation tends to decrease along the length of the sequence and behaves randomly for different FASTQ files. This behavior makes it difficult to predict the nature of the quality scores and hence achieve better compression. We found that the best way to compress this stream is by using a context mixing algorithm, which yielded the best compression ratio for a number of benchmarks. We feed the quality score stream as is to the lpaq8 compressor with option “9” to achieve the best compression ratio.

When conducting experiments both MFCompress and lpaq8 are run in parallel to speed up the process.

### FASTQ data decompression

LFastqC decompresses FASTQ data using the same tools used to compress them in the first place. First, LFastqC regenerates the identifier by decompressing the files of the identifier stream and then merges them to create a single file. Next, lpaq8 and MFCompress decompress both the quality score and DNA sequence in parallel. LFastqC then regenerates the compressed FASTQ data by combining all streams together and uses the quality score file as a reference for the length of each record.

## Results and discussion

We compared our algorithm with two general-purpose compression tools, Gzip [14] and bzip2 [15], as well as other state-of-the-art FASTQ file-compression tools, namely SPRING, LFQC, DSRC2, fqzcomp, SeqSqueeze1 [16], and Quip. FaStore was excluded from this study because it did not work after trying on different platforms. Moreover, a recent study showed that FaStore was outperformed by SPRING on different datasets [13]. For each selected tool, we used their recommended parameters to obtain the best possible compression, as shown in Table 1.

**Table 1:** Compression tools adopted and their parameters.

Algorithm	Parameters
SPRING	-c -i -t 16 / -c -l -i -t 16
LFQC	-
DSRC2	c -m2
Fqzcomp	SOLEXA: -n2 -s7þ -b -q3
	LS454: -n1 -s7þ -b -q2
	SOLiD: -S -n2 -s5þ -q1
SeqSqueeze1	-h 4 1/5 -hs 5 -b 1:3 -b 1:7 -b 1:12 1/10 -bg 0.9 -s 1:2 1/5 -s 1:3 1/10 -ss 10 -sg 0.95
Quip	-
Gzip	-9
Bzip2	-9

Since our tool is a lossless reference-free algorithm, we compared it only with other tools that compress FASTQ data in a lossless manner without using a reference genome. Lossy compression tools are excluded from comparison. Also, tools adopted for comparison are used without any extra information besides the FASTQ file. All the experiments were carried out on a machine running UBUNTU 16.04 64-bit powered by an Intel core i7 processor with 8 GB of RAM. In our comparison, we used publicly available datasets that can be downloaded from the 1,000 Genome Project. These datasets have the same data as those used in [7] as well as in [11]. In addition, three datasets used in [13] as well as four others, such as the PacBio and MinION, are tested on, as shown in Table 2.

**Table 2:** Datasets.

Datasets	Type	Organism	Coverage	Read Length	Size (Mb)
SRR001471	LS454	Homo sapiens	0.07x	188	216
SRR003177	LS454	Homo sapiens	0.27x	564	1196
SRR003186	LS454	Homo sapiens	0.21x	581	886
SRR007215	SOLiD	Homo sapiens	0.07x	25	695
SRR010637	SOLiD	Homo sapiens	0.14x	35	2086
SRR013951	SOLEXA	Homo sapiens	0.89x	76	3190
SRR027520_1	SOLEXA	Homo sapiens	1.19x	76	4808
SRR027520_2	SOLEXA	Homo sapiens	1.19x	76	4808
SRR554369	GAIIx	P.aeruginosa	50x	100	384
SRR327342	GAII	S.cerevisiae	175x	63	2812
SRR1284073	PacBio	E.coli	140x	2942	1302
SRR9046049	PacBio	A. brasilense	136x	3078	2622
SRR8858470	PacBio	Homo sapiens	0.67x	13964	4288
ERR3307082	MinION	C.freundii	367x	4002	3632
ERR637420	MinION	E. coli	118x	6232	264

While selecting datasets, we ensure to include data that were used by previous tools for the sake of a fair comparison, and to include new data for further comparisons. Our datasets incorporate data from different technologies, have different coverages, and different read lengths. The datasets used include three files from LS454, two from SOLiD, as well as five files are from Illumina (three in SOLEXA format, one in GAIIx, and one in GAII format). Our datasets also include three files from PacBio and two from MinION, both widely used. Experimental results revealed the four winners in terms of compression ratio, namely LFastqC, LFQC, SPRING and Fqzcomp, as shown in Table 3. The winning condition is for the compression tool to be able to compress as many different datatypes as possible. Individual winning tools, however, perform better on different datasets, *e*.*g*. LFastqC on LS454, PacBio, and MinION; LFQC on SOLiD dataset; SPRING on Illumina GAIIX and GAII; and Fqzcomp on the Illumina SOLEXA dataset. Although fqzcomp performed the best on Illumina SOLEXA, the results show instability in its performance since it cannot compress the SOLiD datasets due to their color space encoding. Additionally, fqzcomp was not able to compress both PacBio and MinION datasets because it was not able to recognize the file format. Moreover, Quip and SeqSqueeze1were not able to compress both PacBio and MinION datasets except for SRR1284073, which was compressed successfully by Quip. Also noticed was that Quip and SPRING were not able to compress the SOLiD datasets due to the lack of color space encoding support. Our comparison shows that SPRING performs the best when compressing files with short read and medium to high coverage. Fastqz does not work when the read lengths vary so we excluded it from the comparison. The comparisons also revealed the poor performance of the general-purpose compressors when it comes to compression ratio, despite their competitive performance at compression and decompression speed.

**Table 3:** Compression ratios for each tool.

Dataset	Compression Ratio								
	LFastqC	LFQC	SPRING	DSRC2	SeqSqueeze1	Quip	FQZComp	Gzip	Bzip2
SRR001471	**5.29**	5.24	4.58	4.84	5.15	4.47	5.02	3.23	3.93
SRR003177	**5.15**	5.11	4.46	4.60	4.90	4.45	4.77	3.16	3.81
SRR003186	**4.71**	4.64	4.17	4.34	4.63	4.17	4.49	2.97	3.59
SRR007215	6.60	**7.26**	-	6.76	7.07	-	-	4.18	5.20
SRR010637	5.30	**5.59**	-	5.31	5.56	-	-	3.48	4.25
SRR013951	3.46	3.48	3.29	3.39	3.46	3.48	**3.57**	2.40	2.80
SRR027520_1	4.28	4.36	4.14	4.33	4.44	4.48	**4.55**	2.87	3.41
SRR027520_2	4.25	4.27	4.04	4.24	4.35	4.38	**4.45**	2.80	3.33
SRR554369	6.12	5.90	**6.48**	4.32	5.37	4.34	4.94	2.82	3.38
SRR327342	5.90	5.84	**6.45**	4.74	5.64	5.24	6.08	3.07	3.65
SRR1284073	**3.21**	3.20	3.10	-	-	3.10	**-**	2.39	2.82
SRR9046049	**3.09**	**3.09**	2.98	-	-	-	**-**	2.74	2.36
SRR8858470	**3.11**	3.02	2.85	-	-	-	**-**	2.50	2.32
ERR3307082	**2.75**	2.70	2.60	-	-	-	**-**	2.02	2.32
ERR637420	**2.88**	**2.88**	2.81	2.85	-	-	**-**	2.21	2.59

Table 3: Compression ratio is defined as the ratio of the original file size to the compressed file size. Best performance is indicated in bold.

In terms of compression speed, DSRC2 and SPRING show an outstanding performance. Both obtain the best compression speed on different datasets and outperform the other tools in all cases except for two times where they came behind Quip in SRR554369 and SRR1284073. Table 4 summarizes the results.

**Table 4:** Compression speed.

Dataset	Compression Time								
	LFastqC	LFQC	SPRING	DSRC2	SeqSqueeze1	Quip	FQZComp	Gzip	Bzip2
SRR001471	2m00s	3m19s	**0m5**	0m12s	1m45s	0m17s	0m11s	0m41s	0m17s
SRR003177	10m13s	18m04s	**0m22**	0m31s	10m03s	0m39s	1m02s	4m35s	1m35s
SRR003186	7m15s	12m06s	**0m16**	0m17s	7m29s	0m29s	0m59s	3m41s	1m13s
SRR007215	6m18s	6m00s	-	**0m11s**	2m23s	-	-	0m46s	1m10s
SRR010637	21m18s	18m05s	-	**0m41s**	8m21s	-	-	3m30s	3m30s
SRR013951	37m20s	41m04s	0m57s	**0m48s**	25m30s	1m41s	3m06s	8m53s	5m27s
SRR027520_1	44m37s	68m01s	**1m16s**	2m03s	33m44s	2m24s	4m34s	11m17s	7m35s
SRR027520_2	46m42s	59m08s	1m23s	**0m58s**	34m00s	2m22s	4m31s	11m07s	7m37s
SRR554369	5m34s	6m38s	0m15s	0m23s	3m56s	**0m11s**	0m25s	1m12s	0m32s
SRR327342	41m40s	45m0s	2m17s	**0m35s**	20m31s	1m14s	2m20s	6m35s	4m33s
SRR1284073	15m11s	21m21s	1m7s	-	-	**0m38s**	-	3m49s	2m7s
SRR9046049	40m21s	46m52s	**2m56s**	-	-	-	-	4m37s	8m10s
SRR8858470	70m49s	74m47s	**3m35s**	-	-	-	-	7m56s	22m9s
ERR3307082	66m35s	69m23s	**3m01**	-	-	-	-	9m35s	6m53s
ERR637420	3m44s	4m56s	0m24	**0m11s**	-	-	-	0m41s	0m26s

DSRC2 and SPRING also attain the best performance in decompression speed in most cases except for five times where the first place was claimed by either gzip or bzip2. Gzip has the best decompression speed on SRR001471, SRR1284073, and ERR637420. On the other hand, bzip2 has the best decompression speed on SRR9046049 and SRR8858470 as shown in Table 5. Although both DSRC2 and SPRING have demonstrated an impressive speed, neither was able to compress all the different types of data we have in our datasets. SPRING was not able to compress SOLiD. On the other hand, DSRC2 does not support long reads on medium to large sized data. DSRC2 shows an obvious trade-off between the compression ratio and speed. DSRC2, in most cases, was not among the top four in terms of compression ratio except in some cases when other tools failed. This has left us with only four reliable tools that were able to compress all the files within our datasets, which are LFastqC, LFQC, gzip and bzip2. Among these four tools, LFastqC has the best compression ratio on most of the data except for four times when it comes second after LFQC and two times when it shares the same result with LFQC as shown in Table 3. In those cases where LFastqC came after LFQC, we noticed that LFQC performed better in compressing Quality Score, which is largely due to the fact that the back-end compressor ZPAQ performs better on highly randomly generated data than the lpaq8 used in LFastqC. In general, when compressing small to medium sized data, LFastqC performed well both in speed and compression ratio. LFastqC has a better speed and compression ratio when compressing LS454, PacBio, and MinION than LFQC, which has the second-best performance for these types of data. Additionally, LFastqC came second, behind SPRING, in terms of compression ratio on GAIIx and GAII dataset. Nevertheless, LFastqC fell behind when compressing SOLiD and SOLEXA due to the color space encoding in SOLiD dataset and the randomness of the rate of change in correlation between scores in the quality score of SOLEXA.

**Table 5:** Decompression speed.

Dataset	Decompression Speed								
	LFastqC	LFQC	SPRING	DSRC2	SeqSqueeze1	Quip	FQZComp	Gzip	Bzip2
SRR001471	2m16s	3m20s	0m4	0m10s	1m45s	0m47s	0m13s	**0m08s**	0m30s
SRR003177	10m43s	14m48s	**0m16**	0m22s	10m03s	3m40s	1m21s	0m34s	3m00s
SRR003186	7m59s	11m40s	**0m13**	0m20s	7m29s	2m38s	0m58s	0m43s	2m08s
SRR007215_1	6m08s	7m14s	-	**0m11s**	2m23s	-	-	0m23s	1m16s
SRR010637	20m59s	23m28s	-	**0m26s**	8m21s	-	-	1m27s	4m12s
SRR013951_2	35m27s	37m27s	**0m54s**	0m57s	25m30s	9m39s	3m12s	2m34s	8m28s
SRR027520_1	48m27s	56m12s	**1m09s**	2m34s	33m44s	15m38s	5m01s	3m51s	13m57s
SRR027520_2	55m49s	56m59s	**1m09s**	4m01s	34m00s	16m03s	5m24s	4m13s	13m05s
SRR554369_1	5m54s	4m46s	**0m5s**	0m27s	5m16s	1m24s	0m26s	0m6s	0m48
SRR327342_1	40m30s	44m38s	**0m33s**	0m42s	32m12s	8m48s	2m49s	1m56s	5m49s
SRR1284073	16m37s	18m44s	0m31s	**-**	-	2m18s	-	**0m19s**	2m46s
SRR9046049	35m52s	40m29s	2m45s	**-**	-	-	-	6m18s	**1m10s**
SRR8858470	62m59s	68m12s	2m36s	**-**	-	-	-	8m50s	**2m12s**
ERR3307082	56m40s	59m38s	**1m30**	**-**	-	-	-	2m13s	9m16s
ERR637420	2m17s	4m3s	0m12	0m14s	-	-	-	**0m4s**	0m34s

LFastqC memory usage is calculated by summing the memory usage of both compressors, *i*.*e*. lpaq8 and MFcompress since they are running in parallel and the result of their summation is the worst-case scenario. Lpaq8 memory usage is based on argument N, where N can be an integer from 1 to 9. Larger numbers yield a better compression. LFastqC uses lpaq8 with N = 9. The memory usage then can be calculated as follows for both compression and decompression.

$$6+3*2^{9}=1542\;MB$$

On the other hand, LFastqC uses MFcompress with option -3 which uses more memory for better compression. MFcompress with option -3 uses around 2,433 MB for both compression and decompression. This sums up to 4GB in total for each dataset as the worst-case scenario, as shown in Table 6.

**Table 6:** Memory consumption.

Datasets	Size (Mb)	Memory Usage
SRR001471	216	4 GB
SRR003177	1196	4 GB
SRR003186	886	4 GB
SRR007215	695	4 GB
SRR010637	2086	4 GB
SRR013951	3190	4 GB
SRR027520_1	4808	4 GB
SRR027520_2	4808	4 GB
SRR554369	384	4 GB
SRR327342	2812	4 GB
SRR1284073	1302	4 GB
SRR9046049	2622	4 GB
SRR8858470	4288	4 GB
ERR3307082	3632	4 GB
ERR637420	264	4 GB

## Conclusions

We have developed a specialized FASTQ compressor that achieves the best compression ratio on the LS454, PacBio, and MinION datasets with a faster compression and decompression speed than LFQC. LFastqC was compared against two general-purpose compressor and six specialized FASTQ-file compressors. LFastqC outperformed all other tools in eight out of fifteen cases while for the other seven cases the winning spot was shared among FQZComp, LFQC, and SPRING. To sum up, LFastqC was competitive on all datasets due to the elegant preprocessing method and the strength of the two compressors chosen to compress different streams, namely lpaq8 and MFCompress. As of now, LFastqC does not support color space encoding, as well as lpaq8 performance degrades when the quality score stream presents a high degree of randomness, so the performance of LFastqC fell behind the LFQC in some cases. In the future, we will add a feature that supports converting the color space encoding into base space to gain better compression ratios on the SOLiD dataset as well as working on improving the compression ratio of quality score stream.
